# A Polymorphism within the Promoter of the Dopamine Receptor D1 (DRD1 -48A/G) Associates with Impaired Kidney Function in White Hypertensive Patients

**Published:** 2012-01-18

**Authors:** Ersilia Cipolletta, Michele Ciccarelli, Raffaele Izzo, Rosa Finelli, Bruno Trimarco, Guido Iaccarino

**Affiliations:** From the Department of Medicina Clinica, Scienze Cardiovascolari ed Immunologiche, Federico II University, Naples, Italy

## Abstract

Dopamine DRD1 receptor regulates renal function and vascular resistance. It plays a role in the pathogenesis of hypertension in animal models. In humans, the DRD1 gene presents a A-48G polymorphism associated to hypertension in a Japanese population. To explore the role of this polymorphism on blood pressure and renal function in Caucasian hypertensive patients (H), we evaluated the allele frequencies in a populations of 697 H and 100 blood volunteers, and found no difference in the distribution of the alleles between the two groups (AA;AG;GG: 13%;50%;37%; and 12%; 51%;36% respectively). In H, we found a significant difference between AA and GG in serum creatinine (AA: 1.06±.08 mg/dl; GG:0.97±0.02 mg/dl, p<0.03). Treatment restored serum creatinine at levels comparable between genotypes (AA: 0.99±0.03 mg/dl; GG: 0.94±0.02 mg/dl, n.s.). To replicate the finding, in a case control study of 8 AA and 7 GG hypertensive patients matched for age, sex and body mass index, in pharmacological wash out for 30 days, we evaluated serum (Creatinine, Na, Uric Acid, Urea) and urinary (volume/24h, protein/24h, creatinine clearance/24h) biochemistry and renal hemodynamic assessed by ultrasound. Once again, the AA group showed higher serum creatinine, Na, Uric acid and urea, reduced creatinine clearance and a higher level of urinary protein excretion. These changes occurred while no differences were observed in diuresis and renal vascular resistances. In conclusions, the DRD1 A-48G polymorphism identifies a class of H that is prone to hypertension related kidney alterations.

## Introduction

Renal dopamine receptors are important regulator of renal hemodynamic and sodium reuptake, and participate in the pathogenesis of hypertension^1^–^3^.

The dopamine receptors are G-protein coupled receptors subdivided into two families: D1-like (includes D1 and D5) and D2-like (includes D2, D3 and D4) grounded on the stimulation or inhibition of adenylyl cyclase, respectively^4^. D1-like receptors exist on the smooth muscle of renal arteries, the juxtaglomerular apparatus and the renal tubules. D_1_-like receptor agonists increase renal blood flow and glomerular filtration rate, as well as urinary excretion of sodium and water^5^, ^6^. Studies have shown that the natriuretic and diuretic response elicited by D_1_-like receptor agonist involves changes in kidney hemodynamic and direct tubular action^7^. At lower doses, the direct action on renal tubules accounts for the natriuresis and diuresis induced by selective D_1_-like receptor agonists^8^. Since blood pressure is affected by impaired sodium handling, it has been assumed that an aberrant renal dopaminergic system may play a role in the pathogenesis of some forms of hypertension^9^–^11^.

In particular, renal dopamine receptor number and expression have been studied in animal models of hypertension. Watanabe et al. reported that renal cortical dopamine receptor density is diminished in spontaneously hypertensive rats compared with normotensive Wistar-Kyoto rats at 18 weeks of age, but it was similar in both rats at 3 and 7 weeks of age^12^. Renal dopamine receptor density and expression in renal proximal tubules were similar in spontaneously hypertensive rats compared with normotensive Wistar-Kyoto rats, while renal dopamine receptor expression in inner medulla was decreased in spontaneously hypertensive rats compared with control^13^, ^14^. There is defective transduction of the renal dopamine receptor signal in renal proximal tubules of spontaneously hypertensive rats, resulting in decreased inhibition of sodium transport by dopamine^15^. Albrecht et al reported that mice lacking the renal dopamine receptor gene have impaired regulation of renal sodium transport and presented elevated systolic and diastolic blood pressure^9^.

The human dopamine receptor 1 (DRD1) gene has been cloned 16 and localized to chromosome 5 at q35.1, and its gene structure has been described^17^. A polymorphism, A-48G, has been identified at -48 bp of the 5’ untranslated region, which correspond to the DRD1 promoter^18^, ^19^. This mutation harbor the possibility of impaired DRD1 expression, leading to reduced receptor density and signaling. Given the large potentiality of this mutation in the pathogenesis of high blood pressure, the frequency of this polymorphism has been evaluated in a Japanese population and shown to be highly represented among hypertensive patients^20^. On the other hand, large genome wide analysis have failed to confirm association of this gene and hypertension. when The reasons of the interest of this polymorphism not only resides in the pathogenesis of hypertension, which remains a heterogeneous and complex polygenic disease, but also in development of kidney damage related to the hypertensive states. Indeed, hypertension is the second cause of kidney failure, following diabetes. Parameters of kidney damage are used to adjust therapy and patients with similar levels of blood pressure and other anthropometric measurement, have a different evaluation towards organ damage, suggesting that the genetic background play a important role in determining the evolution of this condition. All the above considerations provided the background to verify whether the DRD1 A-48G polymorphism associates to hypertension in a white Caucasian population. Furthermore we pursued exploring the eventual association with biochemical markers of kidney damage.

## Methods

### Subjects

In order to perform an observation of the distribution of the DRD1 variants among hypertensive patients, we selected a study population of 253 essential hypertensive patients referring to the Hypertension Diagnosis and Care Outpatient Clinic of the “Federico II” University of Naples. Anamnesis, clinical data and biochemical features of the patients enrolled in this ambulatory are routinely stored in a computerized database that comprehends over 25000 patients with an average follow-up of 6 years^21^. Patients were randomly selected among those included in the database. Selection criteria were the absence of treatment at first inclusion in the database, presence of essential hypertension, the absence of heart failure, cerebral disease, diabetes and diabetes related target organ damage, concomitant oncological conditions. Blood pressure was measured in supine, sitting and standing posture, according to European guidelines^22^, ^23^. The study was performed in compliance with human study committee regulations of the Federico II University. Written informed consent was obtained from all patients. In another population of 14 patients, we performed a case control study. Patients were selected among those enrolled in the observational study and were homozygous either for the A (AA ) or the G (GG) variant at position −48. Patients were matched for sex, age body mass index and placed in therapeutical wash out for 30 days^24^.

### Biochemical and urinal analysis

At enrolment in the study, blood and urine samples were collected for determination of serum concentration of creatinine, uric, acid, urea, Na, K and analysis of the concentration of creatinine, proteins, Na, K, in the urine collected in 24 hours were measured by standard methods. Based on results, clearance of creatinine was calculated according to the Cockcroft-Gault formula: [(140-Age)*weight in kilograms/]/ serum creatinine*72*(sex correction factor)*BSA/1.73, where sex correction facto is equal 0.85 for women and 1 for men.

### Cardiac, carotid and renal arteries ultrasound

Ultrasounds were performed using a SONOS 5500 (Philips) by two independent trained experienced physicians (NdL, MAER). Intraoperator and interassay variability were 5% and 6%, respectively. Echocardiography was performed using standard technique, with a dedicated ultrasound machine^25^. Cardiac hypertrophy was calculated based on the formula developed by Devereux et al.^26^; cutoffs for left ventricle hypertrophy were considered 110 and 130 g/m^2^ for females and males, respectively^27^.

Carotid artery B-mode ultrasonography was performed with patients in the supine position with the neck extended in mild rotation. The scanning protocol was performed with an ultrasound device (Sonos 5500, Philips) equipped with a 7.5-MHz high-resolution transducer with an axial resolution of 0.1 nm and examinations were recorded on S-VHS videotapes. The maximum arterial intima-media thickness (IMT) in up to 12 arterial walls, including the right and the left, near and far distal common (1 cm) bifurcation, and proximal internal carotid artery was estimated offline with an image processing workstation. Subjects with a maximum IMT of >1.5 mm were considered to have plaque according to previous studies^28^. Renal artery ultrasound is carried out this exam in the early morning after an overnight fast, with the patient supine position. Blood velocity is recorded at the origin of the renal arteries and of intralobar and arcuate vessels. The mean RI ([peack systolic velocity – end-diastolic velocity]/ peack systolic velocity) were calculated 29.

### Analysis of the DRD1 Gene Restriction Fragment Length Polymorphism

From each patient we collected 15 ml of venous blood sample from an antecubital vein for DNA extraction. Genomic DNA was extracted from whole blood samples using a commercially available column extraction kit (Qiagen Midi, Chicago, Illinois, USA). A restriction fragment length polymorphism assay was developed to detect a G allele at nucleotide -48 using the following forward (5-GGC TTT CTG GTG CCC AAG ACA GTG-3) and reverse (5-AGC ACA GAC CAG CGT GTT CCC CA-3) primers^20^. Amplification reaction was carried out in 25 microliters, using 200 ng of genomic DNA, and TAQ DNA polymerase (Qiagen) (annealing: 63°C, 35 cycles) resulting in a 405-bp fragment. The fragment was then digested by DdeI (New England Biolabs) and resolved by electrophoresis over 3% agarose gel^20^. Digestion results in 3 fragments (146, 42, and 217 bp) in the -48G homozygous patients, in 2 fragments (146 and 259 bp) in the -48A homozygous patients conditions, and in the combination of the two profiles in the heterozygous -48A/G patients.

### Statistical Analysis

Data are presented as mean ± SD. Allele frequencies were calculated from the genotypes of all subjects. Significant differences between the total number of alleles on all chromosomes for the hypertensive and normotensive groups were assessed by χ^2^ analysis. Association between genotype and hypertension was evaluated by multiple logistic linear regression analysis. Differences in the clinical data between the subjects with and without hypertension and between subjects with A/A or G/G were assessed by ANOVA followed by Bonferroni’s test. A p value < 0.05 was considered significant.

## Results

### 

#### Demographic and genotypic data

Our population is largely composed of young male patients and in 75% of cases had a family history of hypertension (Table 1). We compared the variant frequencies of the DRD1 gene in hypertensive patients to that of 100 normotensive blood donors and found that they were similar between the two groups and in accord with those reported in other European populations (Table 2).

#### Effects of DRD1 A-48G Genotype on clinical and biochemical characteristics

We then stratified our population based on the DRD1 A-48G polymorphism. There was no effect of the A-48G polymorphisms on anthropometric, hemodynamic and cardiovascular ultrasound parameters (Table 3). Interestingly, patients bearing two copies of the A-48 allele presented with higher level of serum creatinine (Table 3), suggesting a possible interaction of the polymorphism with the kidney function in hypertensive patients.

#### Effects of DRD1 genotype on kidney function and hemodynamics in hypertensives

To further explore whether DRD1 variant can differently interfere with the effects of high blood pressure on kidney function, we selected 15 patients carrying 2 copies of either the A-48 or the G-48 DRD1 alleles. Patients were chosen to match for age, sex, BMI, and blood pressure, and did not present kidney failure or left ventricle hypertrophy. They had similar history of high blood pressure levels and did not present diabetes or dyslipidemia (Table 4). After 30 days of pharmacological wash out, blood pressure increased to a similar extent in both groups. When we looked at serum parameters of kidney function, we confirmed that indeed serum creatinine is higher in the A/A group then the G/G group. Furthermore, also serum Na, nitrogen and uric acid were higher in the A/A group, thus suggesting an effect of this polymorphism on kidney function (Table 5). This is confirmed by the analysis of the 24h urine sample. Indeed, despite similar volumes of urine excretion, calculated creatinine clearance was reduced in the DRD1 A/A patients compared to the G/G (Table 6). Also, microalbuminuria was almost 10 times higher in these patients as compared to the G/G homozygous, indicating a larger kidney damage associated to the hypertensive state depending upon the DRD1 genotype. This phenotype can be related to the hemodynamic effects induced by the DRD1 expressed on the vasculature. We therefore evaluated by ultrasound the renal and kidney artery hemodynamics. Results are illustrated in table 7, and show no differences between hypertensive patients harboring either the A/A or the G/G variant of the DRD1 gene.

## Discussion

The major finding of our work is the identification of the -48A/A variant of the DRD1 gene as a potential risk factor for kidney damage in hypertension. This conclusion relies on the evidences gathered in two different cohorts of patients, using two different experimental approaches. In the first population, using an observational design, in a relatively large cohort of hypertensive patients we report that this polymorphism has same frequency in hypertensive patients and a control population of normotensive blood donors, thus suggesting that this polymorphism, at least in white caucasic does not associate with high blood pressure. More importantly, though, hypertensive patients bearing two copies of the A-48 variant presented a significant increase in serum creatinine levels, which is an indicator of somewhat impaired kidney function. So far, hypertension related organ damage has been considered uniquely a consequence of the exposure to elevated blood pressure levels. Nevertheless, the observation that hypertensive patients with similar anthropometric parameters and blood pressure levels are more prone than others to develop hypertension related target organ damage as well the large familiarity for cerebrovascular events related to high blood pressure levels have provided the necessary background for the investigation of genetic determinants of target organ damage. We have also produced recent evidence that the cardiac damage related to hypertension is indeed determined by a naturally occurring polymorphism of the β2 adrenergic receptor^30^. In our population, similar blood pressure levels as well same anthropometric and clinical features across the DRD1 genotype of our hypertensive patients were found. Therefore, the increase in serum creatinine could find the only explanation in the interference of the DRD1 genotype with the hypertensive status, as indeed confirmed by the multivariate analysis. Furthermore, there are no differences in other parameters of hypertension related organ damage, namely carotid artery atherosclerosis and cardiac size, thus suggesting a specificity that can be related to the kidney abundance of the DRD1 gene expression.

To further detail the nature and characteristics of these kidney alterations, we decided to circumscribe the observation to a limited number of selected patients in which a deeper exploration of kidney function was performed. This part of the study was conducted in selected hypertensive patients that were chosen among the homozygous for the A or the G-48 variant of the DRD1 gene, since no differences were observed between heterozygous and G/G homozygous patients. This case control study, produced evidence that the A/A population is more prone to kidney damage, as suggested by larger microalbuminuria and reduced creatinine clearance. Given the distribution on both vascular smooth muscle cells and tubular epithelium of the DRD1 receptor within the kidney, the altered kidney function can be explained by differences in the kidney hemodynamic or the tubular epithelium sodium reabsorption. Our data support the notion that the A-48ADRD1 mutation leads to an impairment in the kidney filtration function since no differences were observed in the renal artery and parenchymal artery hemodynamic.

Although our data are suggestive that the A-48 mutation leads to an impairment in the function of the DRD1 receptor, the mechanism underlying this alterations are not known. It is possible to speculate that the mutation falls within a regulatory region of the promoter of the gene, but so far there aren’t study available in the literature proving this hypothesis. In this sense, our observation is the first to report an impairment of a physiological function associated with this receptor, therefore supporting the hypothesis that this mutation causes a reduced expression of the DRD1 receptor at the tubular level. Previous studies have explored the impact of this polymorphism on behavior and dependences, since the large abundance of this receptor in the central nervous system and its role in dopamine and serotonine metabolism^31^–^33^. Only another study conducted in a Japanese population report an association of the G/G variant to be more frequent among hypertensives^20^. In our cohort of patients we failed to observe such an association. The different result between the two studies can be well explained by ethnic differences^34^. In particular, among Japanese the A allele is the most frequent, while in our population, the G allele has a larger distribution. The frequency observed in our population is similar to that observed in other Italian and European populations. It is interesting to observe that this distribution appears to be different from that registered for European ancestry populations in other databases, such as Entrez and HAPMAP.

In conclusion, our data support the role of the DRD1 as a genetic marker for hypertension related kidney damage and provide molecular bases for the notion that the hypertension related target organ damage is modified by genotypes. This evidence will lead to development of new therapeutic strategies tailored on the genetic profile of hypertensive patients.

## Figures and Tables

**Table 1:** Demographic, clinical and laboratory characteristics of the study population. Data are expressed as mean ± SD or percentage of the relevant group of patients. SBP: Systolic Blood Pressure; DBP: Diastolic Bloood Pressure

	**n = 736**
**Age (years)**	56.01±11.12
**Gender (M/F %)**	61.7/38.3
**Family history of CV events (%) (before 55 yrs M, 65 yrs F)**	35.7
**Smokers (%)**	42.5
**Ex smokers (%)**	29.6
**No smokers (%)**	25.8
**BMI (Kg/m^2^)**	27.25±3.96
**Blood Glucose (mg/dl)**	96.35±22.75
**Blood Cholesterol (mg/dl)**	209.59±39.35
**Blood Triglycerids (mg/dl)**	137.05±79.17
**HDL Cholesterol (mg/dl)**	48.43±12.45
**LDL Cholesterol (mg/dl)**	131.8±34.78
**Serum Creatinine (mg/dl)**	0.98±0.21
**Urea ( mg/dl)**	37.67±18.49
**Uricic acid (mg/dl)**	5.28±2.56
**Na (mEq/l)**	141.38±3.04
**K (mEq/l)**	4.36±0.43
**SBP (mmHg)**	159.81±22.9
**DBP (mmHg)**	100.94±10.95
**HR (bpm)**	75.85±26.64

**Table 2:** frequencies of DRD1 -48A/G variants among study group and a blood donor cohort.

	**A/A(%)**	**A/G(%)**	**G/G(%)**
**Hypertensives**	**13**	**50**	**37**
**Blood Donors**	**12**	**51**	**36**

**Table 3:** Clinical characteristics of hypertensive population according to DRD1 genotype; SBP: Systolic Blood Pressure; DBP: Diastolic Blood Pressure; LVED: Left Ventricle diastolic diameter; LVMi: Left Ventricle Mass Index; IMT: Intima Media Thickness

DRD1 -48	**A/A**	**A/G**	**G/G**	**P value**
Sex (%)	28/72	39/62	36/64	n.s.
Age (yrs)	47,9±1,3	45,7±1,1	45,6±1,4	n.s.
BMI	26,7±0,5	26,8±0,4	27,2±0,5	n.s.
SBP (mmHg)	142,1±2,7	142,5±1,9	144,8±2,6	n.s.
DBP (mmHg)	92,8±1,6	89,7±1,3	91,3±1,3	n.s.
Serum Creatinine (mg/dl)	1,06±0,08	0,95±0,02	0,97±0,02	**0.023**
Urea (mg/dl)	37,3±2,0	40,3±4,0	34,0±1,3	n.s.
Na (mEq/L)	142±0,7	141±0,4	141±0,5	n.s.
K (mEq/L)	4,4±0,1	4,4±0,0	4,4±0,1	n.s.
LVEDd (mm)	49,5±0,6	50,2±0,3	49,6±0,3	n.s.
Septum Thickness (mm)	10,7±0,2	10,9±0,1	10,8±0,1	n.s.
LVMi	113,6±3,5	119,0±1,9	116,1±2,1	n.s.
Carotid IMTmax	1,4±0,1	1,7±0,1	1,4±0,1	n.s.

F=5.282, p<0.023; t=2.298, p<0.03, ANOVA

**Table 4:** Clinical characteristics of sub study population

	**DRD1 A-48A (n=8)**	**DRD1 G-48G (n=7)**	**P value**
**Age (years)**	46.1±8.3	51.1±4.3	n.s.
**Sex**	4 M 3F	4 M 3F	n.s.
**Body mass index**	24.9±2.6	28.4±3.7	n.s.
**Body Surface Area**	1.8±0.2	1.9±0.2	n.s.
**SBP (mmHg)**	149.6±11.4	153.4±11.4	n.s.
**DBP (mmHg)**	97.8±8.1	99.2±10.7	n.s.
**Heart rate (bpm)**	74.6±9.9	74.3±7.2	n.s.
**Smoke (%)**	14.3	14.3	n.s.
**Diabetes (%)**	0	0	n.s.
**Dyslipidemia (%)**	0	0	n.s.
**History of Elevated Blood pressure (years)**	6.0±5.1	6.4±4.8	n.s

**Table 5:** Serum parameters of renal function in hypertensives according to DRD1 genotype

	**DRD1 A-48A**	**DRD1 G-48G**	**P Value**
**Serum Creatinine (mg/dl)**	0.9±0.06	0.74±0.03	**<0.05**
**Na (mEq/L)**	140±0.6	137±0.9	**<0.05**
**K^+^ (mEq/L)**	4.4±0.3	4.4±0.3	**n.s.**
**Uric Acid (mg/dl)**	5.67±0.3	3.8±0.4	**<0.05**
**Urea (mg/dl)**	37.7±3.1	29.4±1.2	**<0.05**

**Table 6:** Urinary parameters of renal function in hypertensives according to DRD1 genotype

	**DRD1 A-48A**	**DRD1 G-48G**	**P value**
**Diuresis (ml/24 h)**	1582±289	1628±398	**n.s.**
**Protein excretion (mg/24 h)**	128±85	17±16	**n.s.**
**Sodium excretion (mEq/24 h)**	181.6±94.4	170±56.3	**n.s.**
**Potassium excretion (mEq/24 h)**	49.7±9.8	58±22.1	**n.s.**
**Creatinine clearance (ml/min)**	105±6.55	133±13.9	**<0.05**

**Table 7:** Hemodynamic renal parameters in hypertensives according to DRD1 genotype

	**DRD1 A-48A**	**DRD1 G-48G**	**P value**
**PSV proximal renal artery (cm/sec)**	125.28±29.3	112.42±12.9	**n.s.**
**RI proximal renal artery**	0.64±0.03	0.63±0.03	**n.s.**
**PSV intra-parenchimal (cm/sec)**	33.5±10.99	36.48±9.01	**n.s.**
**RI intra-parenchimal**	0.57±0.05	0.56±0.04	**n.s.**

**PSV (peak systolic velocity)**

**RI (resistance index)**
